# A Rare Fungal Infiltration of Lungs in a Healthy Young Girl

**DOI:** 10.1155/2011/917089

**Published:** 2011-09-21

**Authors:** Rajesh Gupta, Nidhi Goel, Anupama Gupta, K. B. Gupta, Uma Chaudhary, Sushma Sood

**Affiliations:** ^1^Department of Tuberculosis & Respiratory Disease, Pt. Bhagwat Dayal Sharma PGIMS, Rohtak, Haryana 124001, India; ^2^Department of Microbiology, Pt. Bhagwat Dayal Sharma PGIMS, Rohtak, Haryana 124001, India; ^3^Department of Physiology, Pt. Bhagwat Dayal Sharma PGIMS, Rohtak, Haryana, 124001, India

## Abstract

*Cunninghamella bertholletiae* is an opportunistic fungal organism found in soil and is a rare cause of human disease. The few reported cases of *C. bertholletiae* have involved immune compromised hosts. We report a case of *C. bertholletiae* in an otherwise healthy patient presenting with persistent high-grade fever and pulmonary infiltration resistant to antibiotics. The organism is isolated through bronchoscopy and responded to broad spectrum antifungal. This is the first case of *C. bertholletiae* reported in the literature in an immune competent individual.

## 1. Introduction

*Cunninghamella bertholletiae* is an opportunistic fungal organism of the order Mucorale. It is found in soil and is a rare cause of human disease [1]. The few reported cases of the fungus have predominantly involved immune compromised hosts and have been associated with very poor outcomes [2–6]. We report a case of *C. bertholletiae* infiltrating the lungs in an otherwise healthy girl presenting with high-grade fever. The patient was treated with broad spectrum antifungal with successful outcome.

## 2. Case Report

**Figure 1:**
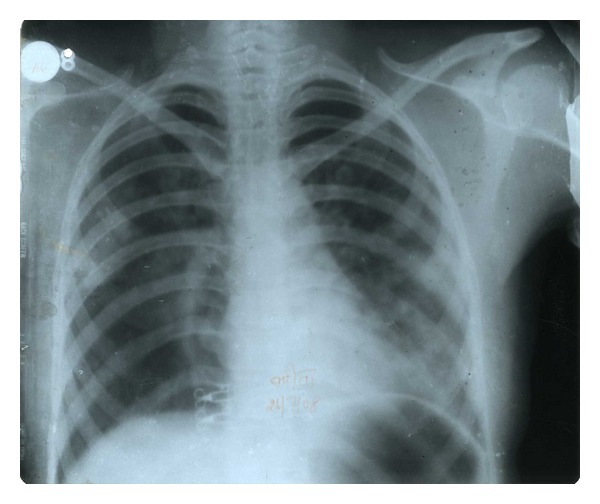
CXR PA showing nonspecific infiltration.

**Figure 2:**
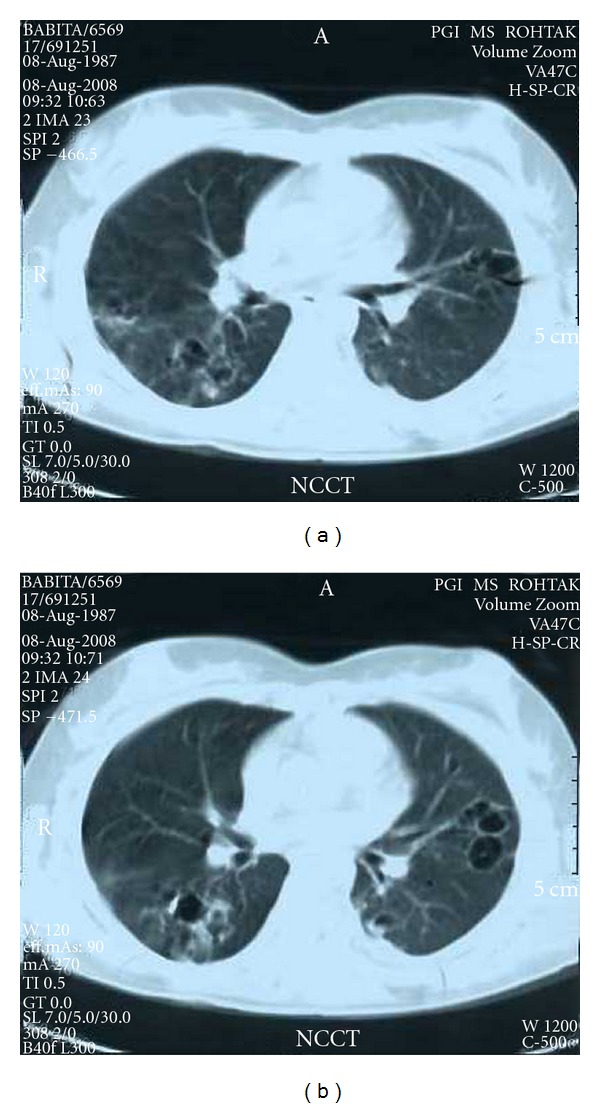
CT sections showing bronchiactatic patches.

**Figure 3:**
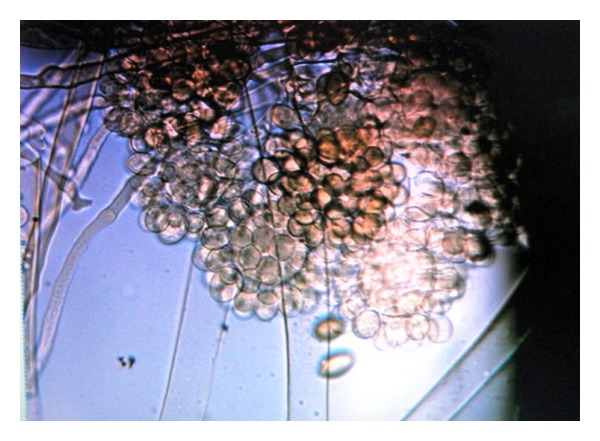
*Cunninghamella bertholletiae*.

An eighteen-year-old unmarried young girl was admitted with fever for fifteen days. She had mild cough and mucoid expectoration with no haemoptysis. The fever was continuous, high grade (varying between 103° to 105°F), with mild chills and rigors off and on (nonspecific). No sore throat, urinary, or bowel problem was reported. Anorexia and weight loss was insignificant. There was no history of tuberculosis or treatment for any major illness in past. History of poisoning was absent. Menstrual history was normal. She was none responding to broad spectrum antibiotics like clarithromycin and amoxicillin-clavulanic acid combination. On examination she was mildly anemic but no cyanosis, lymphadenopathy, jaundice, or any organomegaly could be found. Chest examination revealed only occasional crepts bilaterally. In chest X-ray PA view, low-density peripheral infiltrates were seen bilaterally (Figure 1). On routine investigation, hemoglobin was 10.5 gm%, total leucocytes count was 8000 per cubic mm & differential count was P-59, L-36, M-1, and E-4. Urine routine examination was normal. Widal and PBF for malarial parasite were negative. Sputum for AFB was negative thrice and pyogenic was sterile. Serum HIV was negative. Ultrasonography for abdomen and thorax was normal. On fibro-optic bronchoscopy no lesion could be seen. CT scan sections show peripherally placed bronchiectatic patches (Figure 2). The bronchoalveolar lavage sample on culture revealed unusual fungus *Cunninghamella bertholletiae* (Figure 3). Patient was put on fluconazole empirically at the dose of 150 mg twice a day with a prompt response and was discharged in three days.

## 3. Discussion

*C. bertholletiae* is a rare cause of zygomycosis in humans often associated with trauma and immune suppression. *C. bertholletiae* is the only species of the genus known to cause disease both in humans and animals [1–6]. Humans may be infected through inhalation of airborne fungal spores, however protection against mucor infection is provided by the normal phagocytic and neutrophil function, the exact mechanism of which is not known [7]. Experimental and clinical observations have suggested multiple predisposing factors including acidosis, diabetes mellitus, renal failure, corticosteroids, cytotoxic and antibiotic therapy, leucopenia, hematopoietic malignancies; and depressed phagocytosis. Pulmonary mucormycosis occurs very rarely in healthy individuals. The typical clinical setting consists of an immunologically compromised patient with persistent fever and a progressive pulmonary infiltrate for which no etiological agent has been found [7].

In this case, no immune suppression or predisposing factor was present, otherwise the clinical setting is same for *C. bertholletiae* infection as described above. Patient presented with high-grade fever and lung infiltrations that failed to respond to usual effective antibiotics. The organism was isolated through bronchoscopy. Patient responded to fluconazole promptly within three days and was discharged on long-term antifungal therapy. To our knowledge, this is the first case of pulmonary infiltration of *C. bertholletiae* in an otherwise healthy person reported in the literature. 

 The mucor infections are rare and unknown in healthy individuals and because of difficulty in isolating the organism [6] may go unsuspected and hence undiagnosed. 

 Infection with *C. bertholletiae* is known in immune compromised where it is difficult to treat [2, 3, 5, 6]. In this case, the host is immune competent and it responded to usual antifungal drug promptly. Probably the immunity (the unknown mechanism) acts in synergism with antifungal agent against the organism that happened to anchor the host somehow when seemingly resistance was low.
